# Tumor treating fields inhibit glioblastoma cell migration, invasion and angiogenesis

**DOI:** 10.18632/oncotarget.11372

**Published:** 2016-08-18

**Authors:** Eun Ho Kim, Hyo Sook Song, Seung Hoon Yoo, Myonggeun Yoon

**Affiliations:** ^1^ Korea Institute of Radiological and Medical Sciences, Seoul, Korea; ^2^ Department of Bio-Convergence Engineering, Korea University, Seoul, Korea

**Keywords:** tumor treating fields, glioblastoma multiforme, NF-kB, metastasis, angiogenesis

## Abstract

Treatment with alternating electric fields at an intermediate frequency (100–300 kHz), referred to as tumor treating fields (TTF) therapy, inhibits cancer cell proliferation. In the present study, we demonstrated that TTF application suppressed the metastatic potential of U87 and U373 glioblastoma cell lines via the NF-kB, MAPK and PI3K/AKT signaling pathways. Wound-healing and transwell assays showed that TTF suppressed cell migration and invasion compared with controls. Soft agar and three-dimensional culture assays showed that TTF inhibited both anchorage-dependent (cell proliferation) and anchorage-independent (colony formation) GBM cell growth. TTF dysregulated epithelial-to-mesenchymal transition-related genes, such as vimentin and E-cadherin, which partially accounted for TTF inhibition of cell migration and invasion. We further demonstrated that TTF application suppressed angiogenesis by downregulating VEGF, HIF1α and matrix metalloproteinases 2 and 9. TTF also inhibited NF-kB transcriptional activity. Collectively, our findings show that TTF represents a promising novel anti-invasion and anti-angiogenesis therapeutic strategy for use in GBM patients.

## INTRODUCTION

Gliomas are neuroepithelial tumors that include astrocytomas, oligodendrogliomas, anaplastic astrocytomas and glioblastoma multiforme (GBM) [1, 2]. GBM, which accounts for 50–60% of all gliomas and 20% of all intracranial tumors, is the most common and most malignant glioma [3, 4]. Even with improvements in neurosurgery, chemotherapy and radiotherapy, GBM patient prognoses and survival rates remain extremely poor [5, 6], with a median survival time, two-year survival rate and five-year survival rate of 6–14 months, approximately 10%, and less than 5%, respectively [7, 8]. Aggressive GBM cell migration and invasion lead to a high rate of recurrence [9] and result in the existence of multifocal, subclinical lesions, reducing the efficiency of conventional therapies. New types of therapies that effectively inhibit GBM cell proliferation, migration and invasion will be essential for improving treatment outcomes.

In 2004, tumor treating fields (TTF) therapy, which delivers low-intensity (1–3 V/cm), intermediate-frequency (100–300 kHz) alternating electric fields to tumors, was introduced as a novel cancer treatment modality [10–12]. TTF reportedly causes apoptosis or cell death by inducing mitotic catastrophe and can effectively inhibit the growth of a variety of human and rodent tumor cell lines, with no significant damage to normal cells [10–12]. Previous studies reported that treating recurrent GBM patients with TTF improved overall survival (OS) compared to the standard treatment, with no unexpected adverse effects [13, 14]. Common TTF side effects included medical device site reaction, headache, muscle twitching and skin ulcer. Due to improved clinical outcomes, TTF was approved by the U.S. Food and Drug Administration (FDA) as an alternative to the standard treatment for patients with recurrent GBM, and TTF has received a Communauté Européenne (CE) mark in Europe.

The first randomized study of TTF therapy in recurrent GBM patients suggested that the benefits of TTF alone were not significant compared with conventional chemotherapy. However, a recent randomized clinical trial in patients with newly diagnosed GBM indicated that the use of TTF plus chemotherapy improves median survival compared with chemotherapy alone [15–17]. Previous *in vitro* and clinical studies also reported that TTF improved therapeutic efficacy for multiple cancer types in addition to GBM [10, 15, 16].

That TTF is selectively toxic to proliferating cells via an anti-mitotic mechanism has been widely reported [11], but very few studies have examined the effects of TTF on metastasis. Kirson, *et al.* reported that TTF inhibited solid tumor metastasis to the lungs in two animal models [18], and concluded that TTF may be effective in preventing primary cancer metastatic spread. Still, the underlying mechanisms of TTF action remain unclear. In this study, we applied TTF to GBM cells [19] and investigated the molecular mechanisms of metastasis inhibition by assessing migration, invasion and angiogenesis.

## RESULTS

### GBM cell proliferation inhibition by TTF

**Figure 1:**
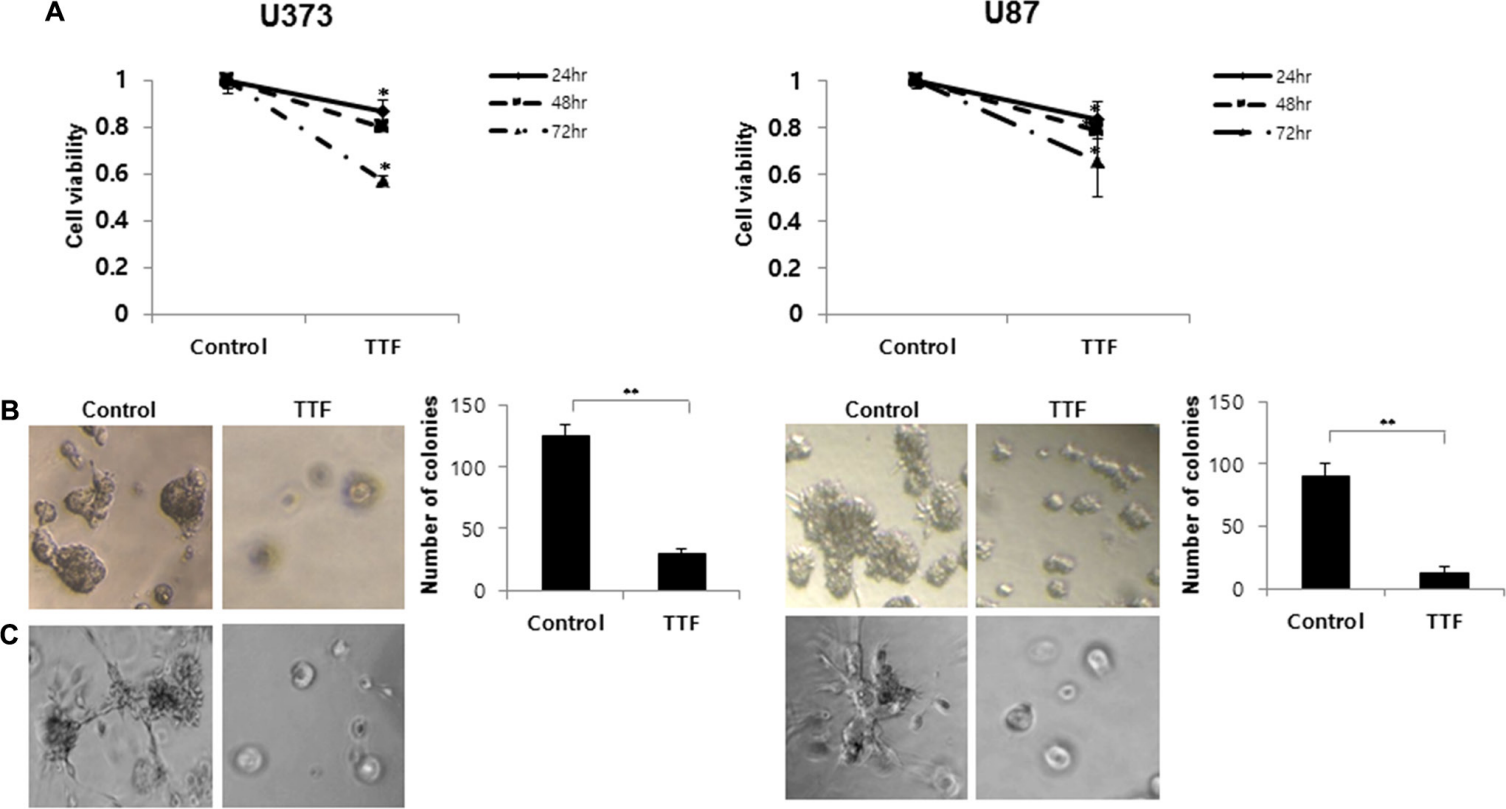
Effect of TTF on the cell proliferation and phenotypic transition of GBM cells (**A**) Cell viability of U373 and U87 cells treated with and without TTF was measured with an MTT assay for the indicated time points. Values represent the means of 3 experiments ± SD; *p < 0.05, **p < 0.001. (**B**) TTF reduces U373 and U87 cell growth in soft agar. Soft agar clonogenic assays were performed with GBM cells after 24hr TTF treatment. Five hundred cells per well were seeded in 12-well plates (3.8 cm^2^) in culture medium containing 0.35% low-melting agarose over a 0.7% agarose base layer and incubated for 14 days. Colonies larger than 100 μm in diameter were counted. Values represent the means of 3 experiments ± SD; *p < 0.05, **p < 0.001. (**C**) Three-dimensional culture model after 24 hr TTF treatment: a thin layer of Matrigel was precoated in 96-well plates as a basement membrane followed by the plating of cells onto the regular medium. TTF-treated cells were observed under an inverted microscope with ×100 magnification.

We examined cell viability at different time points using MTT assays with two GBM cell lines, U373 and U87. To quantitatively measure TTF cell proliferation inhibition, cell death rates were measured at 24, 48 and 72 h after termination of TTF treatment. Cell viability declined over time following TTF treatment. The percentages of viable U373 and U87 cells 24, 48 and 72 h after termination of TTF treatment were 86.5% and 83.3%, 79.5% and 78.6%, and 57.2% and 65.7%, respectively (Figure 1A). This residual effect was reported previously when TTF + chemotherapeutic treatments were applied to human breast carcinoma and human glioma cells [18].

Soft agar assays were performed with GBM cells to assess the effects of TTF *in vitro* (Figure 1B). After 14 days in culture, untreated U373 and U87 cells displayed clonogenic efficiencies of 25% (125.0 ± 8.3 colonies) and 18% (90.0 ± 10.5 colonies), respectively, while TTF-treated cells demonstrated reduced efficiencies of 6.0% (30 ± 3.2 colonies) and 2.4% (12.0 ± 5.3 colonies), respectively. The differences between treated and untreated cells were significant (*p* < 0.001). Additionally, colonies in untreated 3D cultures were larger than those formed by TTF-treated cells (Figure 1C). These results suggested that TTF inhibited both anchorage-dependent (cell proliferation) and anchorage-independent (colony formation) growth of invasive GBM cells.

### TTF suppression of cell migration and invasion

**Figure 2:**
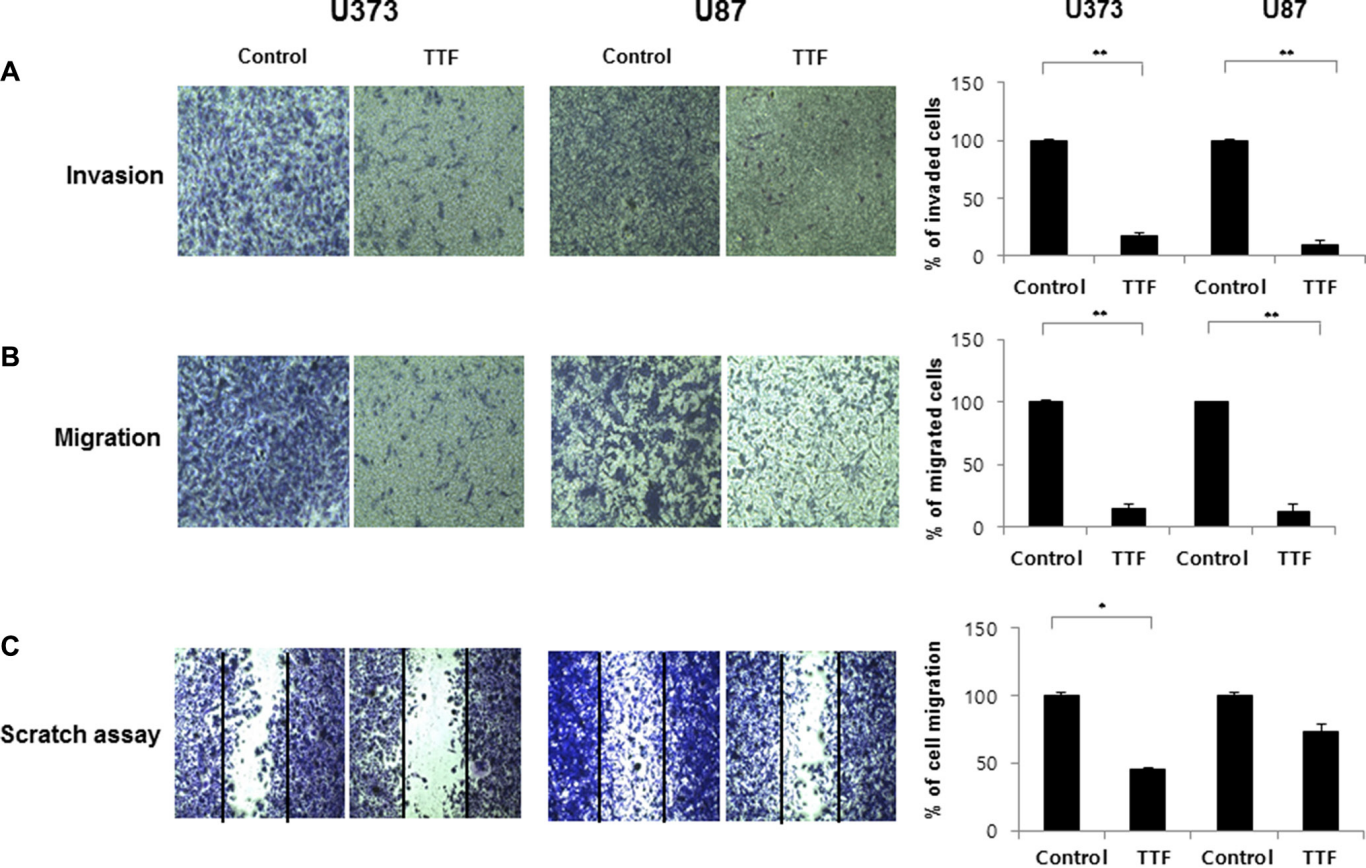
TTF inhibits migration and invasion of GBM cells (**A**, **B**) Tumor cell invasion and migration after 24 hr TTF treatment were examined by transwell chamber assays. The number of invading tumor cells that penetrated through the Matrigel and gelatin was counted using 5 high-intensity fields. Values represent the means of 3 experiments ± SD; *p < 0.05, **p < 0.001. (**C**) GBM cells were treated with TTF for 24 hr TTF treatment and then incubated for 24 h. Then, the cells were scraped with yellow pipette tips for the scratch assay. Values represent the means of 3 experiments ± SD; *p < 0.05, **p < 0.001.

Highly invasive cell growth promotes malignancy in GBM [20]. We investigated the effects of TTF on GBM cell invasion using transwell chamber and wound-healing assays. TTF-treated cells showed reduced migration and invasion in transwell assays compared with controls, using Matrigel [21] and gelatin (Figure 2A–2B). Treatment decreased the percentages of invading/migrating cells by 82/90% and 85/87% for U373 and U87 cells, respectively. Wound healing assay results showed that TTF treatment inhibited cell migration (Figure 2C). TTF application decreased cell migration by 45.6% and 73.6% for U373 and U87 cells, respectively. These results suggest that TTF suppresses GBM cell migration and invasion.

### TTF regulates EMT-related protein and mRNA levels in GBM cells

**Figure 3:**
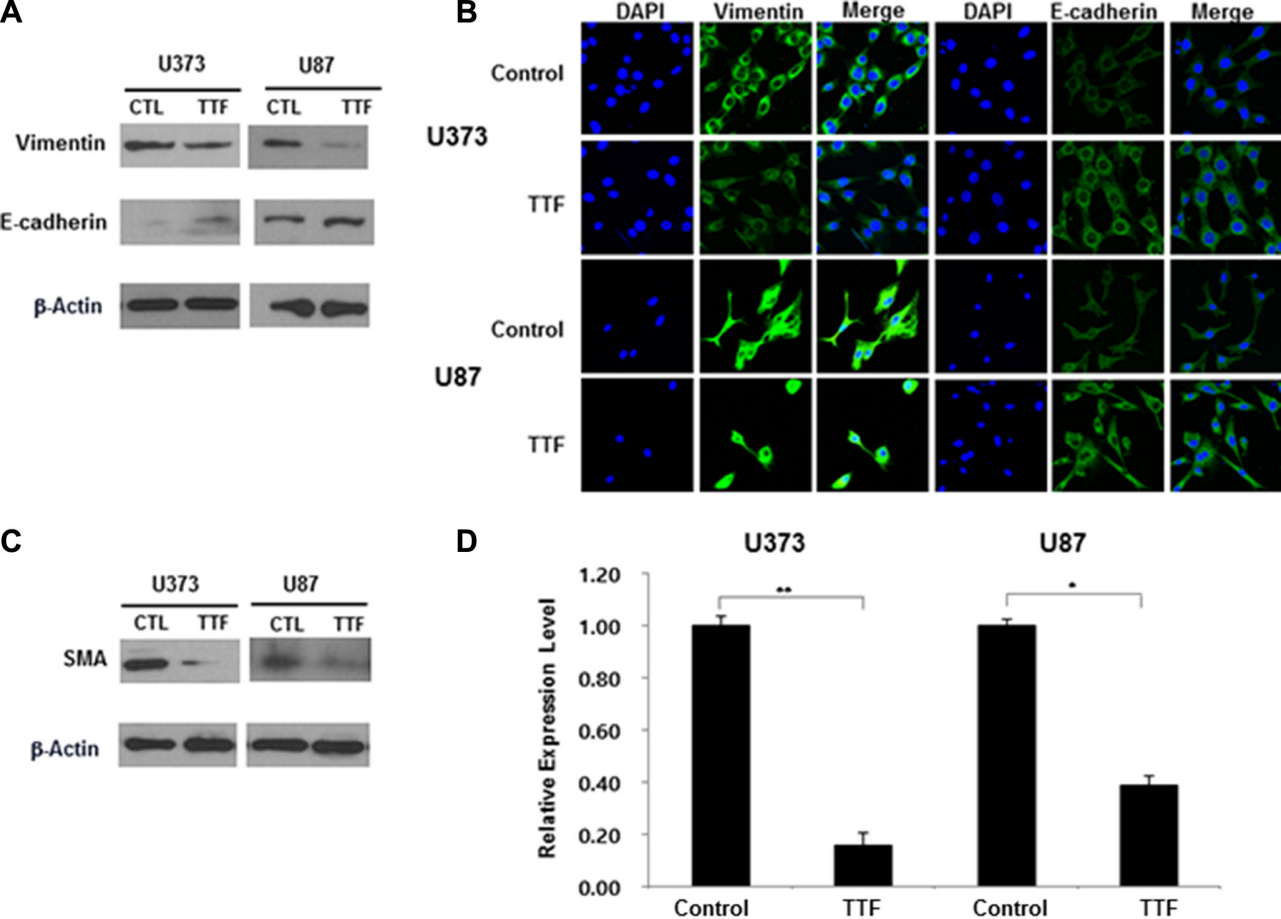
TTF regulates EMT protein levels and EMT-related genes in GBM cells (**A**) Western blotting analysis of E-cadherin and vimentin expression after 24 hr TTF treatment (**B**) Immunocytochemistry staining of E-cadherin and vimentin after 24 hr TTF treatment. In this experiment, GBM cells were treated with TTF for 24 h. (**C**) GBM cells were treated with TTF for 24 h, and western blotting was performed using the antibodies indicated for different time points. (**D**) TTF downregulates SMA mRNA levels as determined by a qRT-PCR array after 24 hr TTF treatment. Values represent the means of 3 experiments ± SD; *p < 0.05, **p < 0.001.

We detected EMT biomarkers in TTF-treated GBM cells by immunofluorescence (IF) analysis, western blotting and qRT-PCR. EMT is essential for cellular invasion and metastasis [22, 23]. Our results indicate that TTF upregulated the epithelial marker E-cadherin and downregulated the expression of the mesenchymal marker, vimentin, compared with controls (Figure 3A–3B). Additional mesenchymal markers, such as smooth muscle actin (SMA), were downregulated in TTF-treated cells (Figure 3C–3D). Dysregulated EMT-related gene expression partially explains the TTF-induced inhibition of GBM cell migration and invasion.

### Inhibition of endothelial cell angiogenesis by TTF

**Figure 4:**
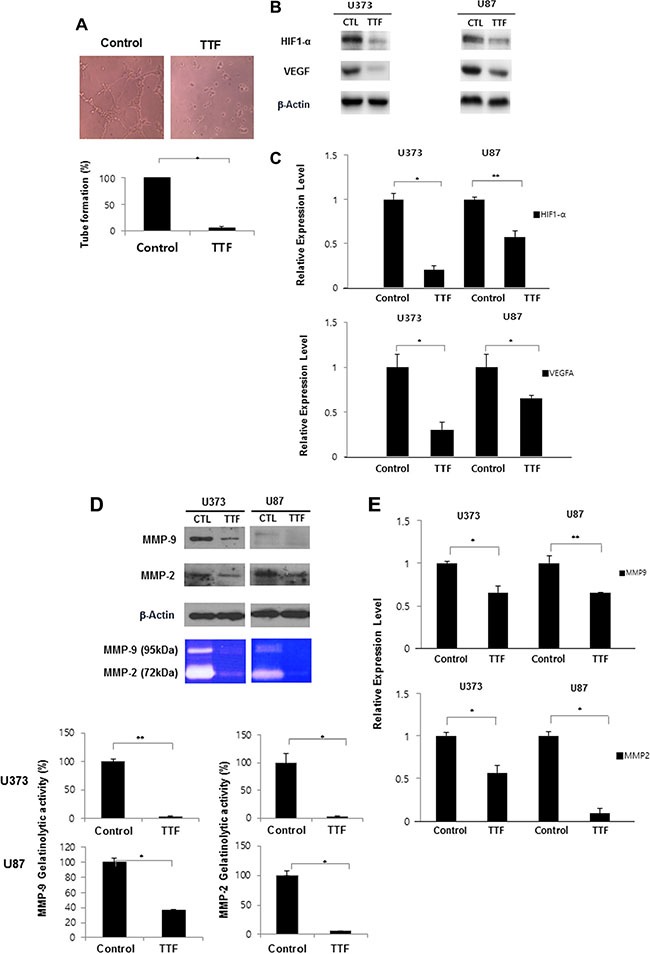
TTF inhibits angiogenesis of endothelial cells through inhibition of MMP-2 and MMP-9 (**A**) Representative photomicrographs of *in vitro* tube formation assays for the Control and TTF treatment groups after 24 hr TTF treatment. Quantitative data for tube formation are expressed as the mean angiogenic score ± SD from three independent experiments. *p < 0.05 and **p < 0.001. (**B**) GBM cells were treated with TTF for 24 h, and western blotting was performed using the antibodies indicated for different time points. (**C**) TTF downregulates HIF1 and VEGF mRNA levels as determined by a qRT-PCR array after 24 hr TTF treatment. Values represent the means of 3 experiments ± SD; *p < 0.05, **p < 0.001. (**D**) MMP-2 and MMP-9 expression was analyzed by western blotting and gelatin zymography after GBM cells were treated TTF for 24 h. Values represent the means of 3 experiments ± SD; *p < 0.05, **p < 0.001. (**E**) TTF downregulates MMP-9 and MMP-2 mRNA levels as determined by a qRT-PCR array after 24 hr TTF treatment. Values represent the means of 3 experiments ± SD; *p < 0.05, **p < 0.001.

Angiogenesis is an essential factor in cancer metastasis [24]. A Matrigel-based tube formation assay using human umbilical vein endothelial cells (HUVECs) showed that TTF suppressed vascular tubule development (Figure 4A). Tube formation was decreased to 5.2% compared to the control 24 h after TTF was applied. Hypoxia-inducible factor 1 (HIF1) activation promotes tumor angiogenesis, growth and metastasis [25], enhancing and regulating the expression of angiogenesis-related genes, such as vascular endothelial growth factor (VEGF) [26, 27]. Our qRT-PCR and western blot analyses revealed that TTF attenuated HIF1α and VEGF expression in TTF-treated cells, suggesting that TTF suppresses angiogenesis via VEGF and/or HIF1α downregulation (Figure 4B–4C).

We performed western blot, zymography and qRT-PCR analyses to detect MMP levels in GBM cells. Following TTF treatment, MMP2 gelatinolytic activity decreased by 97.1% and 97.6% and MMP9 levels decreased by 63% and 94.7% in U373 and U87 cells, respectively, compared to controls (Figure 4D–4E). These results showed that TTF exposure downregulated MMP2 and MMP9 and suggested that TTF may effectively suppress angiogenesis during GBM progression.

### TTF suppresses GBM metastasis through MMP2 and MMP9 inhibition via NF-κB, MAPK and PI3K/AKT signaling

**Figure 5:**
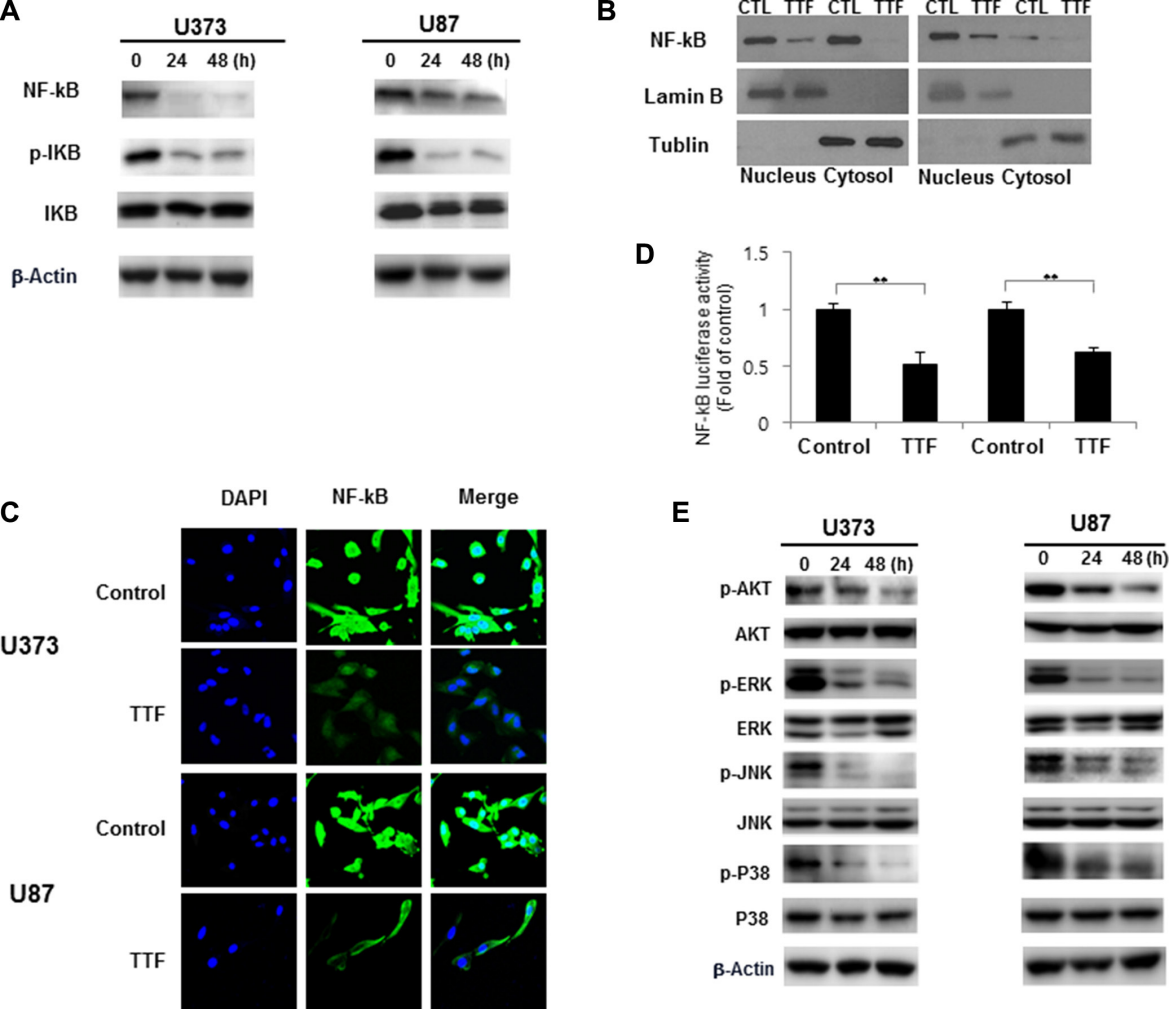
TTF suppresses GBM cancer cell invasion through inhibition of MMP-2 and 9 via the NF-κB pathway (**A**) GBM cells were treated with TTF for 24 h, and western blotting was performed using the antibodies indicated for different time points. (**B**) Cytoplasmic and nuclear fractions of GBM cells were isolated and western blots were performed with anti-NF-kB, anti-α-tubulin and anti-lamin B antibodies after 24 hr TTF treatment. (**C**) GBM cells were treated with TTF for 24 h. For immunofluorescence analysis, cells were stained with an anti-NF-kB antibody and DAPI and observed by confocal microscopy. (**D**) Cells were treated TTF for 24 h and transfected with the NF-kB luciferase reporter construct for 4 h. Luciferase activity was measured using the luciferase assay system. (**E**) GBM cells were treated with TTF for 24 h, and western blotting was performed using the antibodies indicated for different time points.

**Figure 6:**
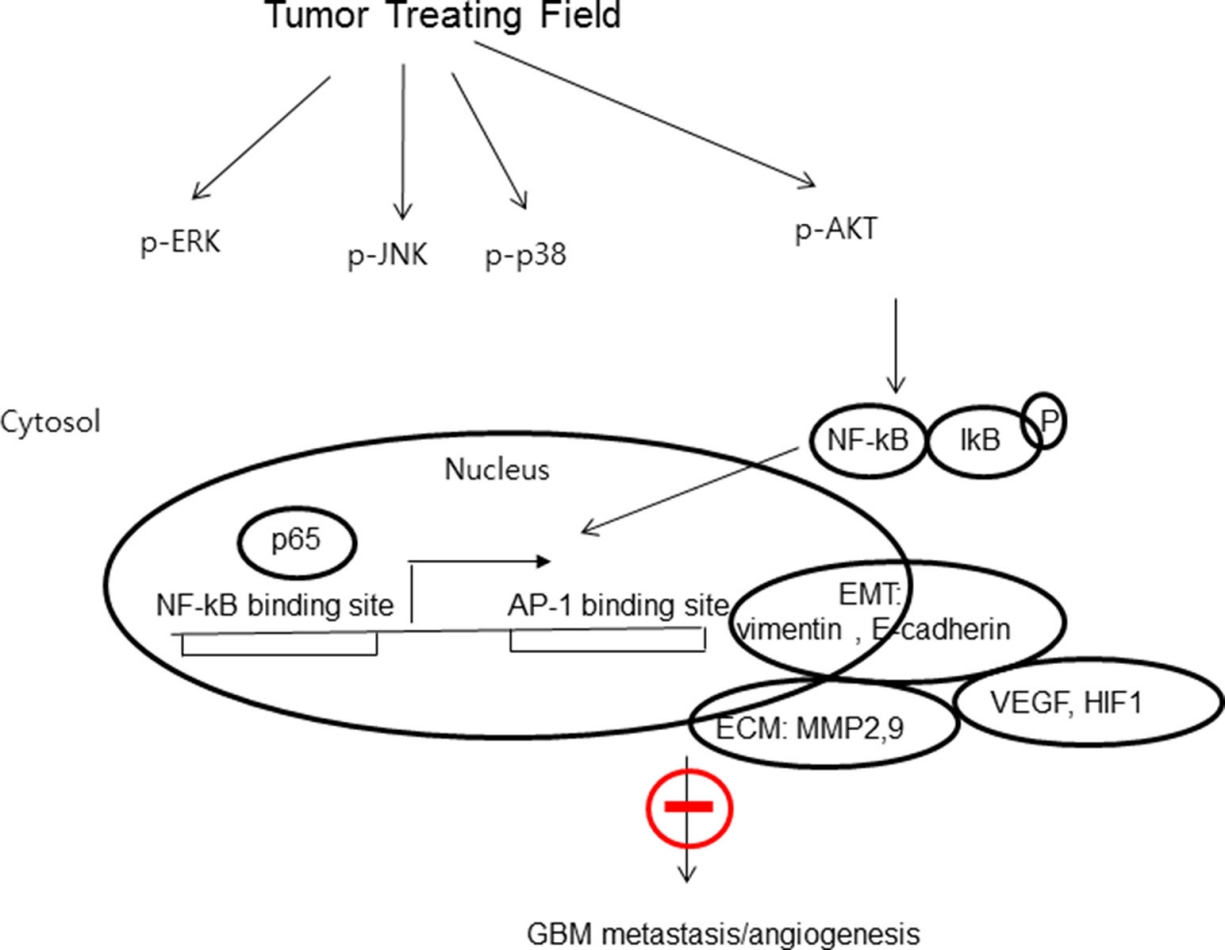
The proposed signaling pathways for TTF-inhibited invasion, migration and angiogenesis in GBM cells Proposed working model for the role of the NF-kB, MAPK and PI3K/AKT signaling pathways in the regulation of TTF-inhibited metastasis of GBM.

NF-κB is an important transcription factor regulating MMP2 and MMP9 expression [28, 29]. Western blotting results showed that TTF efficiently inhibited IkBα phosphorylation in U373 and U87 cells (Figure 5A–5B). TTF also blocked NF-κB p65 (p65) in GBM cells and prevented p65 translocation from the cytoplasm to the nucleus. This finding indicates that TTF inhibited IκBα degradation and NF-κB p65 translocation, and these effects were confirmed by IF assays (Figure 5C). TTF also inhibited NF-κB transcriptional activity (Figure 5D). Furthermore, TTF inhibited p38, ERK, JNK and AKT phosphorylation, while total protein levels of these MAPKs remained unchanged (Figure 5E). Taken together, our results demonstrated that TTF might inhibit GBM cell metastasis through decreased EMT and ECM marker expression, via downregulation of the NF-κB, MAPK and PI3K/AKT signaling pathways (Figure 6).

## DISCUSSION

TTF delivers low-intensity (1–3 V/cm), intermediate-frequency (100–300 kHz) alternating electric fields to tumors and has been utilized as a strategy for GBM therapy in clinics since 2004. In previous reports, TTF demonstrated anti-cancer and anti-proliferative effects in various cell types [11, 15]. TTF appears to inhibit the metastatic spread of solid tumors to the lungs [18]. However, the molecular mechanisms of GBM cell metastasis inhibition by TTF had not been investigated. We designed this *in vitro* study to explore the molecular mechanisms underlying TTF inhibition of tumor cell metastasis. In the current study, TTF inhibited GBM cell invasion, migration and angiogenesis *in vitro*. The biological mechanisms for this effect involve decreased EMT/ECM marker expression and downregulation of NF-κB/MAPK and PI3K/AKT signaling. Inhibition of invasion and migration is an important characteristic of anti-cancer therapeutics [30].

EMT is an important morphogenetic event that promotes cancer cell invasion and metastasis from primary tumors [23, 31, 32]. Although a number of molecular markers are associated with cancer metastasis, one of the most important factors contributing to malignancy is the loss of epithelial differentiation, which is characterized by the loss of E-cadherin–mediated cell-cell junctions and upregulation of mesenchymal markers, including vimentin [33, 34]. Our study showed that TTF increased expression of the epithelial marker, E-cadherin, and decreased expression of the mesenchymal markers, vimentin and SMA (Figure 3). These results suggest that application of TTF to GBM cells blocks tumor cell migration and invasion at least in part through downregulation of EMT-related markers.

In addition to migration and invasion, angiogenesis is another essential factor in cancer progression [24, 35]. In general, tumor growth is dependent on the initiation of new vascularization, extensive cell proliferation and local and distant tumor cell migration [36, 37]. Recent reports suggests that angiogenesis and invasion rely on similar biological mechanisms [38]. Angiogenesis can be regarded as an invasive process in which activated endothelial cells proliferate, adhere to ECM components and migrate [24, 35]. Our results indicate that TTF may also play an important role in suppressing angiogenesis. Overexpression of HIF1α, a transcription factor that immortalizes tumors by inducing expression of genes involved in cell survival, migration and angiogenesis, is related to poor prognosis, increased risk of metastasis and mortality [39]. HIF1α regulates tumor behavior, promoting proliferation, apoptosis and migration; thus, inhibition of the HIF1α pathway may be a promising strategy for inhibiting tumor progression. VEGF, downstream of HIF1α, plays an essential role in blood vessel growth. Many studies have reported on the functions of VEGF and VEGF receptors in angiogenesis, but VEGF is also associated with tumor cell survival, adhesion, migration and invasion [40]. In the present study, TTF decreased HIF1α and VEGF levels in GBM cells.

MMPs are involved in both the angiogenic and invasive processes [41–44]. MMPs degrade ECM molecules, promoting tumor progression and invasion [44]. Of more than 20 known human MMPs, MMP2 and MMP9 appear most important for tumor invasion due to their ability to degrade the ECM and basement membrane [45]. Our western blotting and zymography results indicated that TTF effectively inhibited MMP expression and gelatinolytic activity in GBM cells (Figure 4). The MMP2 and MMP9 promoter region contains cis-regulatory elements, including one NF-κB binding site [46], and NF-κB binding to this site is reportedly closely associated with tumor cell invasion [47]. Thus, we studied the effects of TTF on the NF-κB signaling cascade. NF-κB activation is related to growth, survival, invasion, metastatic potential and drug resistance in many cancers [48]. NF-κB is activated by the phosphorylation-induced degradation of IκBs, which potentializes the dissociation of NF-κB from IκB family proteins. In this study, TTF reduced MMP2 and MMP9 activity via NF-κB downregulation. TTF inhibited IκBα phosphorylation and total NF-κB p65 without influencing total IκBα, and also inhibited NF-κB transcriptional activation (Figure 5A and 5D).

The MAPK and PI3K pathways control proliferation, invasion and migration and, therefore, tumorigenesis [49, 50]. These pathways can be activated by growth factors, inflammatory cytokines, oxidative stress and carcinogens to promote tumor angiogenesis [51, 52]. MAPK pathway inhibition may prevent angiogenesis, proliferation, invasion and migration in various tumors. The PI3K/AKT and MAPK pathways have also been reported to upregulate MMP expression [53, 54]. We evaluated the effect of TTF on markers of the PI3K and MAPK signaling pathways, including AKT, p-AKT, PI3K, p-PI3K, JNK, p-JNK, p38, p-p38, p-ERK and ERK, by western blot analysis. We observed a marked time-dependent decrease in PI3K and MAPK signaling, with a concomitant reduction in cell proliferation and migration. This is the first report elucidating the potential biological mechanisms of TTF in inhibiting GBM cell invasion and migration.

In conclusion, our study demonstrates that TTF can inhibit GBM cell invasion, migration and angiogenesis via downregulation of PI3K/AKT/NF-κB signaling. These inhibitory effects were associated with decreased cell survival, EMT marker expression changes and reduced angiogenesis via decreased HIF1α, VEGF, MMP9 and MMP2 expression. Our results provide a preclinical basis for TTF therapy in GBM patients, although *in vivo* assays addressing invasion, migration and angiogenesis are still needed. Furthermore, tumor initiation and progression are related to cell type and nearby tissue microenvironments; thus, TTF therapy should be further investigated using different cancer cell lines [55, 56]. Collectively, our findings show that TTF represents a promising novel anti-invasion and anti-angiogenesis therapeutic strategy for use in GBM patients.

## MATERIALS AND METHODS

### Experimental setup for electric fields

TTF was generated by a pair of insulated wires (Seoil Electric Wire Co. Ltd; outer diameter, 0.4 mm; polyvinyl chloride insulation thickness, 0.17 mm; dielectric breakdown, 25kV/mm) connected to a function generator (AFG-2112, Good Will Instrument Co., Ltd, Taiwan) and a high-voltage amplifier (A303, A. A. Lab Systems Ltd, Israel) that generated sine-wave signals ranging from 0 to 800 V [19]. To apply the electric field to cell lines, a pair of insulated wires were attached to the bottom of each cell dish, 1 cm from each other [19]. The applied electric field intensity and frequency were 0.9 V/cm and 150 kHz, respectively, for all experiments. We chose 0.9 V/cm because it is very close to the intensity currently used in clinical TTF therapy. As reported previously, the force exerted on a microscopic polarizable organelle in the cell is proportional to the divergence of the electric field squared, which suggests that cell death rate might be non-linearly proportional to the electric field applied [11, 15]. Because the energy density of the electric field is proportional to the electric field applied, the TTF effect (or unit) might be related to energy per unit volume (or unit mass), which is related to the energy density of the electric field. We used MTT assays to determine the optimal TTF conditions. The experimental conditions of 0.9 V/cm and 150 kHz resulted in ∼25% inhibition after 48 h of exposure.

### Cell culture and chemicals

Human glioblastoma U87 and U373 cells were obtained from the Korean Cell Line Bank (Seoul, South Korea). U87 cells were grown in MEM supplemented with 10% FBS, glutamine, HEPES and antibiotics at 37°C in a humidified incubator under 5% CO2. U373 cells were grown in RPMI 1640 medium supplemented with 10% festal bovine serum (FBS), glutamine, HEPES, and antibiotics at 37°C in a humidified incubator under 5% CO2. Anti-vimentin, anti-b-actin, anti-NF-κB, anti-IκB, anti-lamin B, anti-tubulin and anti-p38 antibodies were purchased from Santa Cruz Biotechnology (Santa Cruz, CA, USA). Anti-E-cadherin, anti-SMA, anti-HIF1, anti-VEGF, anti-MMP9, anti-MMP2, anti-p-IκB, anti-p-AKT, anti-AKT, anti-p-ERK, anti-ERK, anti-JNK and anti-p-p38 antibodies were purchased from Cell Signaling Technology (Danvers, MA, USA).

### Soft agar clonogenic assay

Human GBM U373 and U87 cells were treated with TTF for 24 h. Five hundred cells per well were seeded in 24-well plates in culture medium containing 0.35% low-melting agarose over a 0.7% agarose base layer and incubated for 14 days at 37°C in a humidified 5% CO_2_ atmosphere. Colonies larger than 100 μm in diameter were counted under a dissecting microscope. Each cell type was seeded in triplicate, and soft agar assays were repeated three times.

### Three-dimensional (3D) culture system

Human GBM U373 and U87 cells were seeded in 96-well plates at 1 × 10^4^ cells/well. In the 3D culture model, 96-well plates were pre-coated with Matrigel as a basement membrane by adding 40 ul of Matrigel to each well followed by incubation at 37°C for 30 min. Cells were plated onto the gel in appropriate medium, and wells were photographed after 10 days.

### Wound-healing scratch assay and invasion assay

Human GBM cells were seeded onto 6-well plates (Corning) at 2.5 × 10^4^ cells/well in 3 ml medium supplemented with 10% FBS. At 2 days, monolayers were disrupted mechanically using a sterile 200-μl pipette tip. The assay was performed in duplicate. Wells were photographed after 24 h. Cells were then stained with 0.2% crystal violet. Cell migration was monitored using a Nikon Eclipse Ti microscope with a DS-Fi1 camera, and cells were counted using ImageJ software (United States National Institutes of Health, Bethesda, MD, USA).

Invasion was measured *in vitro* using transwell chambers, according to the manufacturer's protocol. Briefly, cells were seeded onto transwell upper chamber membranes at 4 × 10^5^ cells/ml in 150 μl of medium and were left untreated or treated with TTF for 24 h. The medium in the upper chamber was serum-free, while the medium in the lower chamber contained 10% FBS as a chemo-attractant. Cells that passed through Matrigel-coated membranes were stained with a crystal violet solution supplied in the transwell invasion assay kit (Chemicon, Millipore, Billerica, MA, USA) and photographed after 24 h of incubation.

### Immunofluorescence

IF was performed to determine epithelial-to-mesenchymal transition (EMT)-related protein levels in individual cells. Cells were grown on chambered slides 1 day prior to TTF treatment. After TTF exposure for 24 h, cells were fixed. All treatments were performed while cells remained attached to the slides, followed by fixing with 4% paraformaldehyde and permeabilization with 0.2% Triton X-100 in PBS. Detection was performed after blocking the slides in 10% FBS/1% bovine serum albumin for 1 h with FITC-labeled mouse monoclonal antibodies (1:1,000) against vimentin, E-cadherin and NF-kB.

### Western blotting

After TTF treatment, cells were incubated for 24 h, then lysed with RIPA buffer. Proteins were separated by SDS-PAGE and transferred to nitrocellulose membranes, which were blocked with 1% (v/v) non-fat dried milk in Tris-buffered saline with 0.05% Tween 20 and incubated with primary (1:1,000 in 5% bovine serum albumin) and secondary antibodies (1:5,000 in 5% skim milk). Immunoreactive protein bands were visualized by enhanced chemiluminescence (Amersham Biosciences, Little Chalfont, UK) and scanned.

### Quantitative real-time PCR (qRT-PCR)

Total RNA was extracted from individual TTF-treated samples using the TRIzol extraction method as described by the manufacturer (Invitrogen, Carlsbad, CA, USA). First-strand cDNA was synthesized from 1μg of total RNA using the CycleScript RT PreMix (Bioneer, Daejeon, Korea) according to the manufacturer's protocol. Quantitative PCR was conducted using a standard protocol from the SYBR FAST ROX Low qPCR kit (KAPA, Boston, MA, USA). Primer sequences (human) were as follows: α-SMA, Forward: 5′-GATGGTGGGAATGGGACAAA-3′, Reverse: 5′-GC CATGTTCTATCGGGTACTTC-3′; MMP2, Forward: 5′-TGATGGTGTCTGCTGGAAAG-3′, Reverse: 5′-CTAC AGGACAGAGGGACTAGAG-3′; MMP 9, Forward: 5′-T CACTTTCCTGGGTAAGGAGTA-3′, Reverse: 5′-CTGT CAAAGTTCGAGGTGGTAG-3′; VEGFA, Forward: 5′-C AGGACATTGCTGTGCTTTG-3′, Reverse: 5′-CTCAGA AGCAGGTGAGAGTAAG-3′; HIF1α, Forward: 5′-CCA GTTACGTTCCTTCGATCAG-3′, Reverse: 5′-GTAGTG GTGGCATTAGCAGTAG-3′; GAPDH, Forward:5′-AC CCAGAAGACTGTGGATGG-3′, Reverse: 5′-TCTAGA CGGCAGGTCAGGTC-3′. Signals were normalized to GAPDH.

### Matrigel *in vitro* endothelial tube formation assay

Endothelial cell tube formation assays were carried out on Matrigel-coated chamber slides as described previously [21], and results were photographed (Nikon Eclipse Ti microscope with DS-Fi1 camera) at ×40 magnification. Tube formation was quantified by counting the number of connected cells in randomly selected fields at ×400 magnification and dividing that number by the total number of cells in the same field.

### Gelatin zymography

GBM cells were treated with TTF for 24 h, followed by the indicated reagents in serum-free medium. After incubation for the indicated times, the conditioned culture medium and 80% ice-cold acetone (1:4 v/v) were mixed and incubated at –20°C for 2 h. The protein pellet was precipitated following centrifugation at 13,000 rpm for 10 min at 4°C. After washing with 80% ice-cold acetone, pellets were dissolved in deionized water. An aliquot of protein was electrophoresed on an 11% SDS-polyacrylamide gel containing 1 mg/ml gelatin solution. Size-fractionated proteins were renatured twice using 2.5% Triton X-100 for 30 min. After washing with distilled water, the gel was developed in developing solution (50 mM Tris-HCl, 20 mM NaCl, 5 mM CaCl_2_, 0.02% Brij 35, pH 7.6) for the indicated times. For visualization, the gel was stained (0.25% Coomassie Brilliant Blue R-250, 45% ethanol, 10% acetic acid) and then destained (25% ethanol, 5% acetic acid) until background staining disappeared.

### NF-κF luciferase reporter assay

GBM cells were cultured in 12-well plates until 70% confluency, then transfected with the pNF-NFansf plasmid (Beyotime Institute of Biotechnology) using Lipofectamine 2000 (Invitrogen) according to the manufacturer's instructions. After transfection for 4 h, cells were treated with TTF. Luciferase activities were assayed using the Luciferase Assay System (Promega) according to the manufacturer's instructions.

### Statistical analysis

Statistical significance was assessed using Student's *t*-test. Differences were considered significant if *p* ≤ 0.05.

## References

[1] Yin CL, Lv SQ, Chen XY, Guo H (2014). The role of glioma stem cells in glioma tumorigenesis. Front Biosci-Landmrk.

[2] Stupp R, Tonn JC, Brada M, Pentheroudakis G, Grp EGW (2010). High-grade malignant glioma: ESMO Clinical Practice Guidelines for diagnosis, treatment and follow-up. Ann Oncol.

[3] Lehrer S, Green S, Ramanathan L, Rosenzweig K, Labombardi V (2012). No Consistent Relationship of Glioblastoma Incidence and Cytomegalovirus Seropositivity in Whites, Blacks, and Hispanics. Anticancer Res.

[4] Crocetti E, Trama A, Stiller C, Caldarella A, Soffietti R, Jaal J, Weber DC, Ricardi U, Slowinski J, Brandes A, Grp RW (2012). Epidemiology of glial and non-glial brain tumours in Europe. Eur J Cancer.

[5] Stupp R, Hegi ME, Mason WP, van den Bent MJ, Taphoorn MJB, Janzer RC, Ludwin SK, Allgeier A, Fisher B, Belanger K, Hau P, Brandes AA, Gijtenbeek J (2009). Effects of radiotherapy with concomitant and adjuvant temozolomide versus radiotherapy alone on survival in glioblastoma in a randomised phase III study: 5-year analysis of the EORTC-NCIC trial. Lancet Oncol.

[6] Lima FRS, Kahn SA, Soletti RC, Biasoli D, Alves T, da Fonseca ACC, Garcia C, Romao L, Brito J, Holanda-Afonso R, Faria J, Borges H, Moura-Neto V (2012). Glioblastoma: Therapeutic challenges, what lies ahead. Bba-Rev Cancer.

[7] Schwartzbaum JA, Fisher JL, Aldape KD, Wrensch M (2006). Epidemiology and molecular pathology of glioma. Nat Clin Pract Neuro.

[8] Siebzehnrubl FA, Reynolds BA, Vescovi A, Steindler DA, Deleyrolle LP (2011). The origins of glioma: E Pluribus Unum?. Glia.

[9] Sanai N, Berger MS (2008). Glioma extent of resection and its impact on patient outcome. Neurosurgery.

[10] Davies AM, Weinberg U, Palti Y (2013). Tumor treating fields: a new frontier in cancer therapy. Ann N Y Acad Sci.

[11] Kirson ED, Gurvich Z, Schneiderman R, Dekel E, Itzhaki A, Wasserman Y, Schatzberger R, Palti Y (2004). Disruption of cancer cell replication by alternating electric fields. Cancer Res.

[12] Pless M, Weinberg U (2011). Tumor treating fields: concept, evidence and future. Expert Opin Inv Drug.

[13] De Bonis P, Doglietto F, Anile C, Pompucci A, Mangiola A (2012). Electric fields for the treatment of glioblastoma. Expert Rev Neurother.

[14] Rulseh AM, Keller J, Klener J, Sroubek J, Dbaly V, Syrucek M, Tovarys F, Vymazal J (2012). Long-term survival of patients suffering from glioblastoma multiforme treated with tumor-treating fields. World J Surg Oncol.

[15] Kirson ED, Dbaly V, Tovarys F, Vymazal J, Soustiel JF, Itzhaki A, Mordechovich D, Steinberg-Shapira S, Gurvich Z, Schneiderman R, Wasserman Y, Salzberg M, Ryffel B (2007). Alternating electric fields arrest cell proliferation in animal tumor models and human brain tumors. P Natl Acad Sci USA.

[16] Kirson ED, Schneiderman RS, Dbaly V, Tovarys F, Vymazal J, Itzhaki A, Mordechovich D, Gurvich Z, Shmueli E, Goldsher D, Wasserman Y, Palti Y (2009). Chemotherapeutic treatment efficacy and sensitivity are increased by adjuvant alternating electric fields (TTFields). BMC Med Phys.

[17] Stupp R, Wong E, Scott C, Taillibert S, Kanner A, Kesari S, Ram Z (2014). Ef. Interim Analysis of the Ef-14 Trial: A Prospective, Multi-Center Trial of Novottf-100a Together with Temozolomide Compared to Temozolomide Alone in Patients with Newly Diagnosed Gbm. Neuro-Oncology.

[18] Kirson ED, Giladi M, Gurvich Z, Itzhaki A, Mordechovich D, Schneiderman RS, Wasserman Y, Ryffel B, Goldsher D, Palti Y (2009). Alternating electric fields (TTFields) inhibit metastatic spread of solid tumors to the lungs. Clin Exp Metastas.

[19] Jeong H, Sung J, Oh SI, Jeong S, Koh EK, Hong S, Yoon M (2014). Inhibition of brain tumor cell proliferation by alternating electric fields. Appl Phys Lett.

[20] Wong ET, Hess KR, Gleason MJ, Jaeckle KA, Kyritsis AP, Prados MD, Levin VA, Yung WKA (1999). Outcomes and prognostic factors in recurrent glioma patients enrolled onto phase II clinical trials. J Clin Oncol.

[21] Kumar P, Benedict R, Urzua F, Fischbach C, Mooney D, Polverini P (2005). Combination treatment significantly enhances the efficacy of antitumor therapy by preferentially targeting angiogenesis. Lab Invest.

[22] Yang J, Weinberg RA (2008). Epithelial-mesenchymal transition: At the crossroads of development and tumor metastasis. Dev Cell.

[23] De Craene B, Berx G (2013). Regulatory networks defining EMT during cancer initiation and progression. Nat Rev Cancer.

[24] Nishida N, Yano H, Nishida T, Kamura T, Kojiro M (2006). Angiogenesis in cancer. Vasc Health Risk Manag.

[25] Yoon JW, Jun HS (2002). Recent advances in insulin gene therapy for type 1 diabetes. Trends Mol Med.

[26] Hellwig-Burgel T, Stiehl DP, Wagner AE, Metzen E, Jelkmann W (2005). Review: hypoxia-inducible factor-1 (HIF-1) : a novel transcription factor in immune reactions. J Interferon Cytokine Res.

[27] Weijts BGMW, Bakker WJ, Cornelissen PWA, Liang KH, Schaftenaar FH, Westendorp B, de Wolf CACMT, Paciejewska M, Scheele CLGJ, Kent L, Leone G, Schulte-Merker S, de Bruin A (2012). E2F7 and E2F8 promote angiogenesis through transcriptional activation of VEGFA in cooperation with HIF1. Embo J.

[28] Lin CW, Hou WC, Shen SC, Juan SH, Ko CH, Wang LM, Chen YC (2008). Quercetin inhibition of tumor invasion via suppressing PKC delta/ERK/AP-1-dependent matrix metalloproteinase-9 activation in breast carcinoma cells. Carcinogenesis.

[29] Woo JH, Lim JH, Kim YH, Suh SI, Min DS, Chang JS, Lee YH, Park JW, Kwon TK (2004). Resveratrol inhibits phorbol myristate acetate-induced matrix metalloproteinase-9 expression by inhibiting JNK and PKC delta signal transduction. Oncogene.

[30] Friedl P, Wolf K (2003). Tumour-cell invasion and migration: Diversity and escape mechanisms. Nat Rev Cancer.

[31] Kalluri R, Weinberg RA (2009). The basics of epithelial-mesenchymal transition. J Clin Invest.

[32] Thiery JP, Sleeman JP (2006). Complex networks orchestrate epithelial-mesenchymal transitions. Nat Rev Mol Cell Biol.

[33] Tsai JH, Yang J (2013). Epithelial-mesenchymal plasticity in carcinoma metastasis. Genes Dev.

[34] Voulgari A, Pintzas A (2009). Epithelial-mesenchymal transition in cancer metastasis: mechanisms, markers and strategies to overcome drug resistance in the clinic. Biochim Biophys Acta.

[35] Folkman J, Shing Y (1992). Angiogenesis. J Biol Chem.

[36] Hanahan D, Folkman J (1996). Patterns and emerging mechanisms of the angiogenic switch during tumorigenesis. Cell.

[37] Liotta LA, Steeg PS, Stetler-Stevenson WG (1991). Cancer metastasis and angiogenesis: an imbalance of positive and negative regulation. Cell.

[38] Skobe M, Rockwell P, Goldstein N, Vosseler S, Fusenig NE (1997). Halting angiogenesis suppresses carcinoma cell invasion. Nat Med.

[39] Thiery JP, Acloque H, Huang RY, Nieto MA (2009). Epithelial-mesenchymal transitions in development and disease. Cell.

[40] Perrot-Applanat M, Di Benedetto M (2012). Autocrine functions of VEGF in breast tumor cells: adhesion, survival, migration and invasion. Cell Adh Migr.

[41] Haas TL, Milkiewicz M, Davis SJ, Zhou AL, Egginton S, Brown MD, Madri JA, Hudlicka O (2000). Matrix metalloproteinase activity is required for activity-induced angiogenesis in rat skeletal muscle. Am J Physiol-Heart C.

[42] Rundhaug JE (2003). Matrix metalloproteinases, angiogenesis, and cancer - Commentary re: A. C. Lockhart et al., reduction of wound angiogenesis in patients treated with BMS-275291, a broad spectrum matrix metalloproteinase inhibitor. Clin. Cancer Res., 9:00–00, 2003. Clin Cancer Res.

[43] Chambers AF, Matrisian LM (1997). Changing views of the role of matrix metalloproteinases in metastasis. J Natl Cancer I.

[44] Egeblad M, Werb Z (2002). New functions for the matrix metalloproteinases in cancer progression. Nat Rev Cancer.

[45] Clark IA, Swingler TE, Sampieri CL, Edwards DR (2008). The regulation of matrix metalloproteinases and their inhibitors. Int J Biochem Cell B.

[46] Aggarwal BB (2004). Nuclear factor-kappa-B: The enemy within. Cancer Cell.

[47] Eberhardt W, Huwiler A, Beck KF, Walpen S, Pfeilschifter J (2000). Amplification of IL-1 beta-induced matrix metalloproteinase-9 expression by superoxide in rat glomerular mesangial cells is mediated by increased activities of NF-kappa B and activating protein-1 and involves activation of the mitogen-activated protein kinase pathways. J Immunol.

[48] Kang H, Lee M, Choi KC, Shin DM, Ko J, Jang SW (2012). N-(4-hydroxyphenyl)retinamide inhibits breast cancer cell invasion through suppressing NF-KB activation and inhibiting matrix metalloproteinase-9 expression. J Cell Biochem.

[49] Li B, Yang YH, Jiang SD, Ni BB, Chen K, Jiang LS (2012). Adenovirus-mediated overexpression of BMP-9 inhibits human osteosarcoma cell growth and migration through downregulation of the PI3K/AKT pathway. Int J Oncol.

[50] Rasmussen N, Rathmell WK (2011). Looking beyond inhibition of VEGF/mTOR: emerging targets for renal cell carcinoma drug development. Curr Clin Pharmacol.

[51] Van Waes C (2007). Nuclear factor-kappaB in development, prevention, and therapy of cancer. Clin Cancer Res.

[52] Peng L, Liu A, Shen Y, Xu HZ, Yang SZ, Ying XZ, Liao W, Liu HX, Lin ZQ, Chen QY, Cheng SW, Shen WD (2013). Antitumor and anti-angiogenesis effects of thymoquinone on osteosarcoma through the NF-kappa B pathway. Oncol Rep.

[53] Guo LY, Li YM, Qiao L, Liu T, Du YY, Zhang JQ, He WT, Zhao YX, He DQ (2012). Notch2 regulates matrix metallopeptidase 9 via PI3K/AKT signaling in human gastric carcinoma cell MKN-45. World J Gastroentero.

[54] Kang MH, Oh SC, Lee HJ, Kang HN, Kim JL, Kim JS, Yoo YA (2011). Metastatic function of BMP-2 in gastric cancer cells: The role of PI3K/AKT, MAPK, the NF-kappa B pathway, and MMP-9 expression. Exp Cell Res.

[55] Barcellos-Hoff MH, Ravani SA (2000). Irradiated mammary gland stroma promotes the expression of tumorigenic potential by unirradiated epithelial cells. Cancer Res.

[56] Wiseman BS, Werb Z (2002). Development - Stromal effects on mammary gland development and breast cancer. Science.

